# Prevalence, predictors, and outcome of pulmonary mucormycosis in COVID-19 associated rhino orbital mucormycosis in a tertiary care center in South India

**DOI:** 10.22034/cmm.2023.345154.1486

**Published:** 2023-09

**Authors:** Karthigeyan Thanjavur Sethuraman, Jayaraj Athimanjeri Thiruvengadam, Abinaya Ravichandran, Santhi Thoppappatty Sengottaiyan

**Affiliations:** Institute of Internal Medicine, Madras Medical College and Rajiv Gandhi Government General Hospital, Chennai, Tamil Nadu, India

**Keywords:** COVID-19-associated mucormycosis, CT chest, Mucormycosis, Pulmonary mucormycosis, Severe COVID-19 pneumonia

## Abstract

**Background and Purpose:**

India witnessed an explosive rise in mucormycosis following COVID-19 infection. Pulmonary mucormycosis closely followed rhino orbital mucormycosis as the most common presentation. The need for advanced resources and lack of clinical suspicion for COVID-19-associated pulmonary mucormycosis led to widespread underdiagnosis and poor response to late therapy. The present study aimed to assess the prevalence of pulmonary mucormycosis in COVID-19-associated rhino-orbital mucormycosis using non-invasive techniques, such as sputum microscopy and chest imaging.

**Materials and Methods:**

A prospective observational study was conducted at the Institute of Internal Medicine, Rajiv Gandhi Government General Hospital in Chennai, India between June 2021 and July 2021. All hospitalized patients with proven rhino orbital mucormycosis with or without cerebral involvement within three months of confirmed COVID-19 infection who had clinical symptoms compatible with pulmonary mucormycosis were included in this study. These patients were screened for probable and possible COVID-19-associated pulmonary mucormycosis using computed tomography (CT) chest imaging and sputum microscopy within 48 h of hospital admission.

**Results:**

Based on the findings, 8 (16%) out of 50 patients with rhino-orbital mucormycosis, had associated possible or probable pulmonary mucormycosis. All 8 patients were diabetics and had characteristic CT chest findings while only half of them had positive sputum microscopy. A higher prevalence of probably disseminated COVID-19-associated mucormycosis was noted among 51-60-year-old males with the use of corticosteroids and oxygen for COVID-19 therapy. The mortality rate was 100% in probably disseminated mucormycosis, 50% in possible disseminated mucormycosis, and only 9.5% in isolated rhino-orbital mucormycosis.

**Conclusion:**

Non-invasive and feasible methods, such as sputum microscopy and chest imaging can be considered for early screening and intensive management of probably disseminated mucormycosis to improve prognosis.

## Introduction

The COVID-19 pandemic paved the way for an unanticipated fungal epidemic in the form of mucormycosis, particularly in India [ 1 ]. Superadded infections were infrequently reported in the early phases of the pandemic; however, several reports of fungal infections linked to COVID-19 infections surfaced during the second wave of this disease [ 2 ]. Over 45,432 cases and 4,252 deaths due to mucormycosis have been documented in India as of July 15, 2021, either among COVID-19-infected patients or among patients who had recovered from COVID-19 [ 3 ]. 

Six major forms of invasive mucormycosis have been described. These include cutaneous, gastrointestinal, disseminated, rhino-orbito-cerebral, pulmonary, and uncommon forms, like endocarditis, osteomyelitis, peritonitis, and renal infection [ 4 ]. Disseminated disease, defined by the involvement of two or more non-contiguous sites, has a devastatingly high mortality rate amounting to almost 90% [ 5 ].

Traditionally, various predisposing factors have been linked to the site of mucormycosis involvement. While uncontrolled diabetic patients are more prone to develop rhino-orbital mucormycosis, patients with hematological malignancies and transplant recipients are more prone to develop pulmonary mucormycosis [ 6 ]. The most common manifestation of the COVID-19-associated mucormycosis (CAM) outbreak was rhino-orbital cerebral mucormycosis, which was followed by pulmonary mucormycosis [ 2 ]. 

The exact prevalence of pulmonary mucormycosis was most likely underestimated during the pandemic due to the lack of routine diagnostic facilities and clinical guidelines [ 7 ]. According to the Delphi consensus statement from the Fungal Infection Study Forum and Academy of Pulmonary Sciences, India, pulmonary mucormycosis diagnosed either at the same time as, or within 3 months of, confirmed COVID-19 was agreed upon as the entry criterion for diagnosing COVID-19-associated pulmonary mucormycosis [ 8 ].

While diabetes remains the cornerstone for the development of CAM, other factors, such as hypoxia, metabolic acidosis, glucocorticoid use, and increased ferritins in COVID-19 have been implicated as predisposing factors [ 2 ].

With congruent clinico-radiological features, mucormycosis is likely when *Mucorales* are isolated from sputum or endotracheal aspirate. An initial investigation could be a sputum examination as it is a non-invasive procedure. As bronchoscopy-obtained respiratory samples are more indicative of the disease site, their diagnostic yield may be high [ 9 ]. 

Diagnostic imaging is essential for early detection. Although computed tomography (CT) chest could be used as an initial method, the demonstration of angio-invasiveness in lung tissue samples continues to be the cornerstone of diagnosis. However, it is important to note that invasive tissue sampling is often delayed or not feasible due to the critical nature of the underlying illness.

This study was undertaken to assess the prevalence of probable and possible pulmonary mucormycosis in patients presenting with COVID-19-associated rhino-orbital mucormycosis using easily available non-invasive techniques, such as sputum microscopy and CT chest. Furthermore, the prognosis of patients having probably disseminated CAM in comparison to rhino-orbital mucormycosis was estimated, and probable predictors of poor outcomes were analyzed.

## Materials and Methods

### Study population

This prospective observational study was conducted at the Institute of Internal Medicine, Rajiv Gandhi Government General Hospital in Chennai, India between June 2021 and July 2021. All hospitalized patients of any gender aged 18 years and above with microbiologically or histologically proven rhino orbital mucormycosis with or without cerebral involvement within 3 months of confirmed COVID-19 infection who had clinical symptoms compatible with pulmonary mucormycosis were included in this study.

The presence of brownish/black sputum or hemoptysis was considered a highly suggestive clinical symptom, while fever, worsening or productive cough, chest pain, or worsening respiratory symptoms in patients with COVID-19 were considered suggestive of pulmonary mucormycosis.

Pregnant and breastfeeding females were excluded. The hospital Ethics Committee approved this study (Institution Ethics Committee Code No. 23062021) and informed written consent for participation in the study was obtained from all the participants.

### Study subjects and definitions

Diagnosis of rhino orbital mucormycosis with or without cerebral involvement was made based on the presence of consistent radiological and clinical findings, along with the identification of fungi in the tissue of the patient by either isolation of *Mucorales* or direct microscopic visualization of broad ribbon-like aseptate hyphae. Patients who tested positive for severe acute respiratory syndrome Coronavirus 2 (SARS-COV2) RNA in respiratory specimens by reverse transcription polymerase chain reaction or those with a positive rapid antigen test either at the time of present hospital admission or within three months from the date of present admission were diagnosed with COVID-19. In this study, CAM was defined as the occurrence of proven mucormycosis in COVID-19 patients within three months of diagnosis of COVID-19 infection.

Pulmonary mucormycosis was categorized into three groups based on the level of evidence for diagnosis [ 8 ]. Histopathology or cytology showing aseptate hyphae or culture obtained by a sterile procedure from pleural fluid or lung showing growth of *Mucorales* was considered proven COVID-19-Associated Pulmonary Mucormycosis (CAPM). Presence of compatible clinical features, risk factors, suitable imaging along with demonstration of aseptate hyphae with or without positive culture, in a sample from the lower respiratory tract including bronchoalveolar lavage, non-bronchoscopic bronchial lavage, bronchial washings, bronchial brushing, endotracheal aspirates, and sputum was diagnosed as probable CAPM [ 8 ]. Presence of compatible clinical features, uncontrolled diabetes, and prolonged glucocorticoid therapy with highly suggestive radiology in the absence of a definite alternative diagnosis was considered possible CAPM [ 8 ].

As rhino-orbital mucormycosis with or without cerebral involvement and pulmonary mucormycosis were the most common forms of CAM, the definition of probably disseminated CAM in the present study was confined to the involvement of rhino-orbital mucormycosis with or without cerebral involvement along with possible or probable CAPM.

Given the critical nature of the illness, a resource-limited setting, and the need to ensure accessibility, only non-invasive methods were chosen for early screening of CAPM in the form of sputum analysis and CT chest imaging. Hence, patients with compatible clinical and diagnostic features were categorized only into probable or possible CAPM. Diagnosis of proven CAPM using invasive histopathological tissue was beyond the scope of the present study.

### Methodology

Based on the inclusion criteria, the patients enrolled in the study were screened for probable and possible CAPM using CT chest imaging and sputum microscopy within 48 h of hospital admission. 

Presence of thick-walled cavity, reversed halo sign, large consolidation or necrotizing pneumonia, mycotic aneurysm, bird’s nest sign, multiple large nodules (nodules >1 cm), or serial imaging showing cavity with an air-fluid level in a patient with compatible clinical features were considered to be highly suggestive of CAPM. Conventional microscopy using potassium hydroxide mount with or without calcofluor stain (manufactured by HiMedia Laboratories Private Limited, India) was used to analyze sputum specimens.

All patients received liposomal Amphotericin B with dose modifications as needed. The induction therapy duration depended on the patient tolerance of amphotericin B infusion. Additionally, surgical debridement for rhino-orbital mucormycosis was performed on patients as required. It should be mentioned that anticoagulant therapy was provided for one patient with additional pulmonary embolism. Furthermore, oral triazoles were given at the time of discharge.

Details regarding patient demographics, underlying conditions, and clinical course of COVID-19, including therapeutics, oxygen, or ventilator support were documented. The interrelationship was analyzed using SPSS software version 27.

## Results

During the study period, 50 patients with symptoms compatible with suspected pulmonary mucormycosis were admitted with COVID-19-associated rhino-orbital mucormycosis with or without cerebral involvement. All 50 patients were included in the study. The mean age of the study population was 54.18 ± 10.66 years with a male: female ratio of 2.12:1. Only seven patients had no known comorbidities, while the majority of the study population suffered from one or more comorbid illnesses specifically diabetes mellitus (86%).

With respect to treatment during COVID-19 infection, 64% of the patients had received oxygen therapy for a minimum of one week using any modality (nasal cannula, face mask, non-rebreather mask, high flow nasal oxygen, or mechanical ventilation). Parenteral steroids had been administered to 70% of the patients ranging from a minimum duration of one week to a maximum duration of 15 days depending on the protocol of the respective institution for COVID-19 treatment.

**Figure 1:**
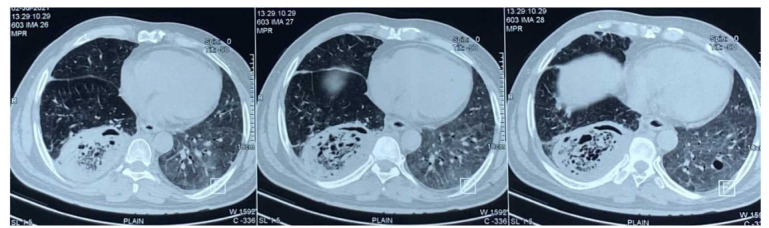
A 56-year-old male was admitted as a case of COVID-19-associated mucormycosis with respiratory symptoms. Computed tomography chest plain axial section shows evidence of a thick-walled cavity with central ground glass opacities and peripheral consolidation in the right lower lobe (bird’s nest sign) suggestive of pulmonary mucormycosis.

**Figure 2:**
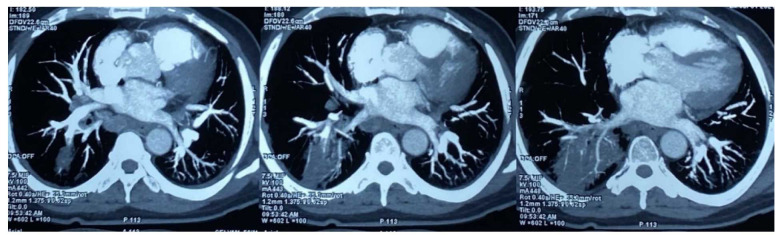
A 62-year-old male admitted for COVID-19-associated mucormycosis with sudden worsening of respiratory symptoms. Computed tomography pulmonary angiogram axial section shows central intraluminal thrombosis with complete occlusion of the posterior descending branch of the right pulmonary artery with consolidation changes in the posterobasal segment of the right lower lobe.

All 50 patients were subjected to sputum analysis and CT chest imaging. Conventional sputum microscopy using potassium hydroxide mount with or without calcofluor white stain demonstrated broad aseptate hyaline hyphae suggestive of *Mucorales* in only four samples. The CT chest with highly suggestive findings of CAPM (Figure 1) was reported in 16% of the patients with one patient additionally having complete thrombosis of the posterobasal branch of the right pulmonary artery (Figure 2). Hence, based on the above-mentioned findings, four patients were diagnosed with probable CAPM, and four patients were diagnosed with possible CAPM. The prevalence of probably disseminated CAM, that is, the presence of rhino-orbital mucormycosis with or without cerebral involvement along with probable CAPM or possible CAPM was 8% each in the study population.

In total, 6 out of the 10 deaths in the study population happened in patients with probably disseminated CAM. The mortality rate was 100% in disseminated mucormycosis with probable CAPM and 50% in disseminated mucormycosis with possible CAPM in contrast to only 9.5% in CAM patients without lung involvement. Hence, this association between mortality and the presence of probable or possible pulmonary mucormycosis in CAM patients was found to be statistically significant (*P*-value= 0.001).

Though the risk of probably disseminated CAM was found to be higher among diabetics and patients with other comorbidities, the association was not statistically significant. Furthermore, all four patients with probable CAPM (100%) and three patients with possible CAPM (75%) were treated with parenteral steroids during COVID-19 therapy. Moreover, oxygen therapy was provided for all patients with probable CAPM and half of the patients with possible CAPM as a part of COVID-19 treatment. However, these associations were not statistically significant.

## Discussion

The COVID-19 pandemic increased the susceptibility to fungal and bacterial infections [ 10 ]. Though the most frequently reported COVID-19-associated fungal infections were due to Aspergillus and Candida [ 11 ], the highly underreported mucormycosis has a fatal prognosis. Several studies conducted in the Indian subcontinent have revealed rhino-orbital mucormycosis as the most common form of CAM followed by pulmonary mucormycosis [ 2 , 3 ]. It is highly probable that due to the deficiency of routine diagnostic facilities and clinical guidelines, the exact burden of disseminated mucormycosis was largely underestimated during the pandemic.

The present study aimed to estimate the prevalence of probably disseminated CAM using easily available diagnostic methods. However, the definition of probably disseminated CAM was confined to include only the two most common forms of CAM, that is rhino-orbital mucormycosis with or without cerebral involvement and pulmonary mucormycosis.

Prevalence of probably disseminated CAM was estimated to be 16% of the study population. In a literature review from 18 countries, 20 cases of pulmonary mucormycosis were reported out of 80 cases of COVID-19-associated mucormycosis with the rest mostly being rhino-orbital mucormycosis [ 12 ]. In a systematic review performed by Pal et al. on 99 cases of CAM, rhino-orbital mucormycosis was most the common infection (42%), followed by rhino-orbito-cerebral mucormycosis (24%) while pulmonary mucormycosis was observed in 10 patients (10%) [ 13 ]. However, literature regarding the exact prevalence of disseminated CAM is lacking.

Conventional sputum microscopy was positive in only 4 out of 50 patients with compatible clinical symptoms and 8 patients with characteristic CT chest imaging. Although the Delphi consensus statement from the Fungal Infection Study Forum and Academy of Pulmonary Sciences, India [ 8 ] advocates the use of sputum examination as a non-invasive initial diagnostic method, the yield is low. In addition, they recommend the use of respiratory samples obtained using bronchoscopy as they are more representative of the disease site. 

In a study involving 24 patients with confirmed pulmonary mucormycosis, three patients were diagnosed through sputum examination while nine patients were diagnosed through bronchoscopy, which emphasizes the poor yield of sputum examination similar to the findings of the present study [ 14 ].

Characteristic CT chest findings, such as the presence of a thick-walled cavity with internal septations, bird’s nest sign, and reversed halo sign were observed in eight patients. Further difficulties in the diagnosis of CAPM arise when lung abnormalities associated with COVID-19 are visible on imaging. It has been demonstrated that COVID-19 causes both the halo sign and reverse halo sign; in different series, the prevalence of both signs ranges from 0% to 18% [ 15 , 16 ]. It is possible to distinguish between COVID-19 and CAPM with the use of serial imaging. In COVID-19, the halo sign and reverse halo sign have a tendency to improve with time [ 17 ]; however, in pulmonary mucormycosis, cavitation is the typical course [ 18 , 19 ]. Consequently, the course of the illness, clinical setting (uncontrolled diabetes, persistent or recent onset fever, hemoptysis, or productive cough), and timing all aid in distinguishing between acute COVID-19 and CAPM.

Presence of uncontrolled diabetes, especially diabetic ketoacidosis, use of steroid therapy, and oxygenation support have been significantly associated with increased risk of CAM [ 2 , 20 , 21 ]. However, these factors do not necessarily increase the risk of probably disseminated CAM as evidenced by the present study.

The outcomes were devastatingly poor in patients with probably disseminated CAM mounting to 100% in patients with probable CAPM and 50% in patients with possible CAPM. Accordingly, the mortality rate of probably disseminated CAM was extremely high in comparison to that of CAM without pulmonary involvement (9.5%) despite similar protocol-based management in all patients.

Treatment decisions for these patients must be made on an individual basis, preferably following consultation with a multidisciplinary team that includes radiologists, clinicians, and thoracic surgeons. The Delphi Consensus advises surgical debridement for CAPM for all surgically resectable lesions [ 8 ]. However, factors, such as extensive invasion of mediastinal structures or hilar vessels and the general condition of the patient may limit aggressive treatment options.

Antifungal medications that are effective against *Mucorales* should be used in the treatment of all patients with confirmed or probable CAPM. The panel of experts advised against the routine treatment of patients with possible CAPM as their treatment needs to be individualized [ 9 ]. However, it is important to note that patients with a delay in initiation of therapy have poor prognosis, usually turning fatal [ 22 ].

### Limitations of the study

This study was conducted at the peak of the COVID-19 pandemic which limited the use of invasive tissue diagnosis for confirmation of CAPM due to various factors, such as limited resources and the unstable general condition of the patient. Moreover, the sensitivity and specificity of sputum microscopy and CT chest for screening of CAPM could not be assessed due to a lack of comparison with the gold standard technique.

## Conclusion

The CAM is a critical problem complicating COVID-19 pneumonia, especially in developing nations, like India, which is also the diabetes capital of the world. Stringent measures must be adopted to prevent CAM, such as aggressive control of risk factors, including diabetic control and judicious use of steroids and oxygen therapy. Among various types of CAM, probably disseminated CAM is associated with high mortality rates. Owing to poor clinical suspicion, nonspecific clinical picture overlapping with COVID-19 symptoms and multiple barriers to diagnosis, probably disseminated CAM with pulmonary involvement remains highly underdiagnosed. Non-invasive feasible diagnostic modalities, such as CT chest and sputum microscopy should be strongly considered for screening of patients with CAM with persistent respiratory symptoms, especially in resource-poor settings, for early initiation of intensive care and aggressive monitoring to improve outcomes.
